# Supplementary material for: Reprogramming of human peripheral blood mononuclear cells into induced mesenchymal stromal cells using non-integrating vectors
Source: Commun Biol. 2023 Apr 11;6:393. doi: 10.1038/s42003-023-04737-x (PMC10090171; doi:10.1038/s42003-023-04737-x)
Supplement: Supplementary file 2 — Supplementary Information [file 42003_2023_4737_MOESM2_ESM.pdf]

Reprogramming of human peripheral blood mononuclear cells into induced mesenchymal stromal cells using non-integrating vectors

Wanqiu Chen[1], Chenguang Wang[1,2], Zhi-Xue Yang[3,4], Feng Zhang[3,4], Wei Wen[3,4], Christoph Schaniel[5], Xianqiang Mi[2], Matthew Bock[6], Xiao-Bing Zhang[3], Hongyu Qiu[7*], Charles Wang[1,8*]*

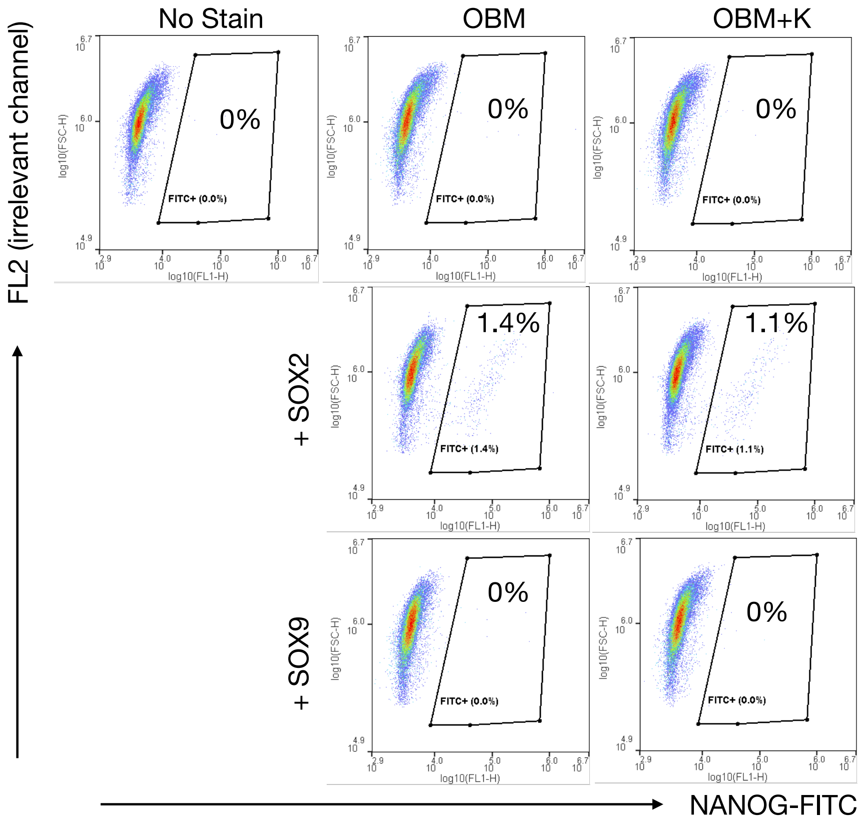

Supplementary Figure 1. Flow cytometric analysis of iMSCs showing that reprogramming in the presence of SOX2 resulted in 1-2% reprogrammed cells that were NANOG positive while replacing SOX2 with SOX9 generated no detectable NANOG^{+} cells.

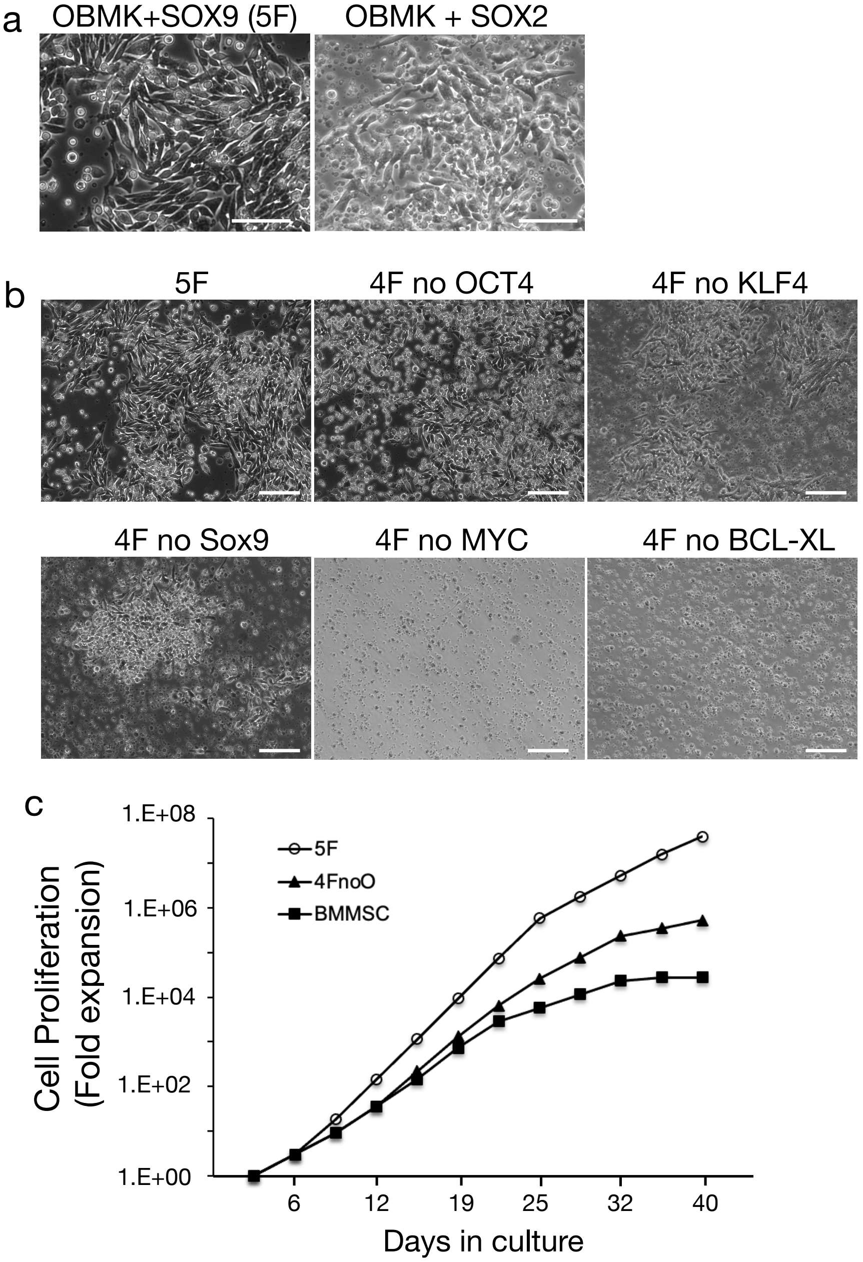

Supplementary Figure 2. Representative images of human adult PBMCs transfected with different combinations of non-integrating episomal vectors at the day 7 post-transfection. (**a**) PBMCs transfected with four reprogramming factors (OCT4, BCL-XL, MYC, and KLF4) as well as either SOX2 or SOX9. SOX9-reprogrammed cells displayed more spindle-like morphology. Scale bar represents 100 μm. (**b**) PBMCs transfected with five reprogramming factors (OCT4, BCL-XL, MYC, KLF4, and SOX9), and any combination of four reprogramming factors by omitting a single factor to assess the essentiality of the five factors. Scale bar represents 200 μm. (**c**) Proliferation of 5F iMSCs, 4FnoO iMSCs, and bone marrow derived human MSCs (BMMSC).

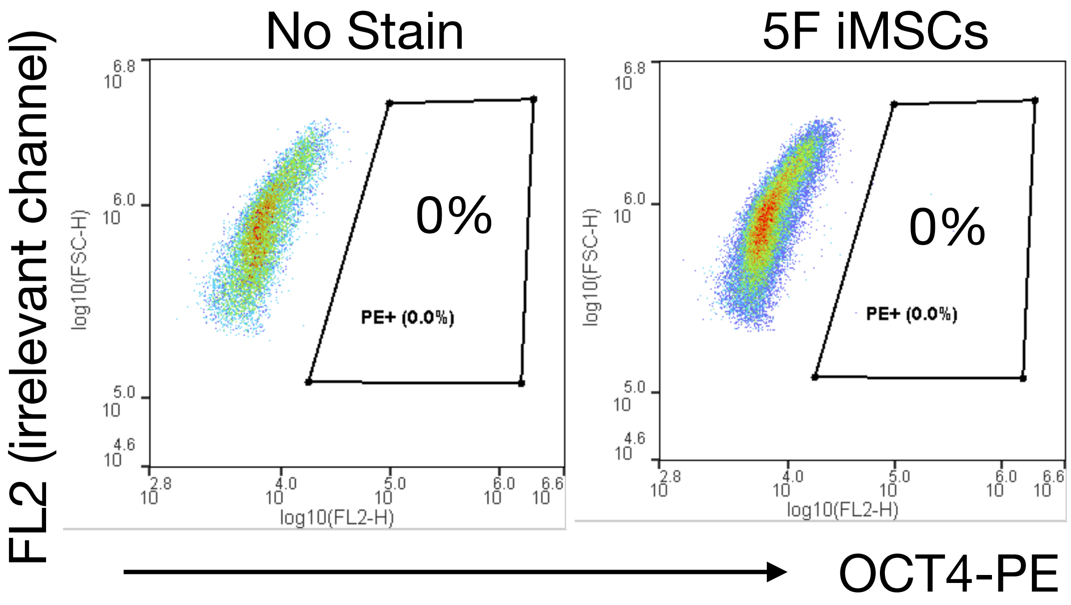

Supplementary Figure 3. Flow cytometric analysis of iMSCs at one month after nucleofection showing that no detectable OCT4^{+} cells.

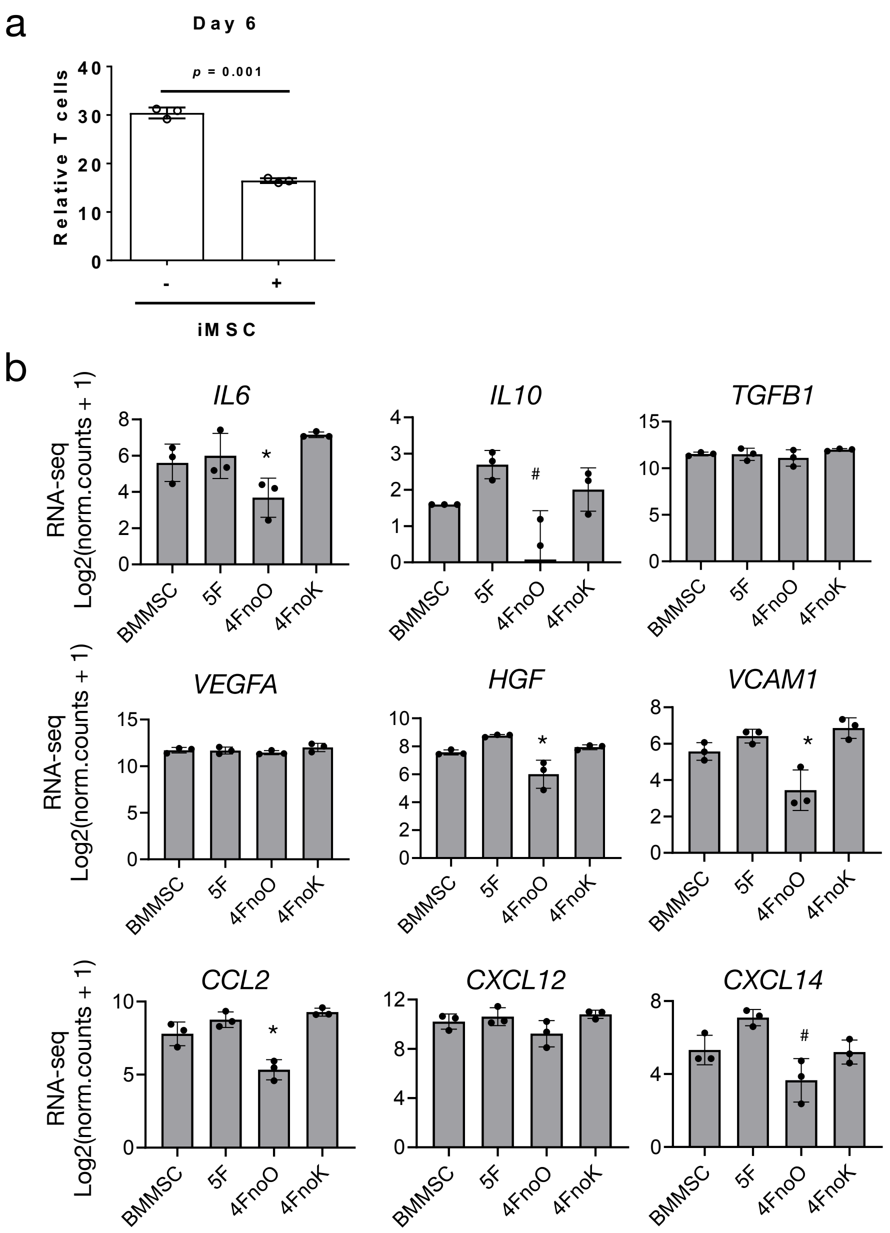

Supplementary Figure 4. Immuno-modulating function and RNA-seq gene expression of immunomodulatory factors of iMSCs. (a) iMSCs significantly inhibited T-cell proliferation after 6 days co-culture with PBMCs. **(b)** The bar plotting showing RNA-seq gene expression values of the representative genes of immunomodulatory cytokines, chemokines, and soluble factors secreted by MSCs. Human primary MSCs, i.e., bone marrow-derived MSCs (BMMSC) derived RNA-seq gene expression data[23] was used as control. RNA-seq gene expression levels are shown as $\log_2()$ normalized read counts. n=3 in each group. $^{*}P < 0.05$ indicates the statistically significance using BMMSC as control group; $^{\#}P < 0.05$ indicates the statistically significance using the 5F MSC as control group; error bars indicate standard deviation.

PBMC digital karyotyping

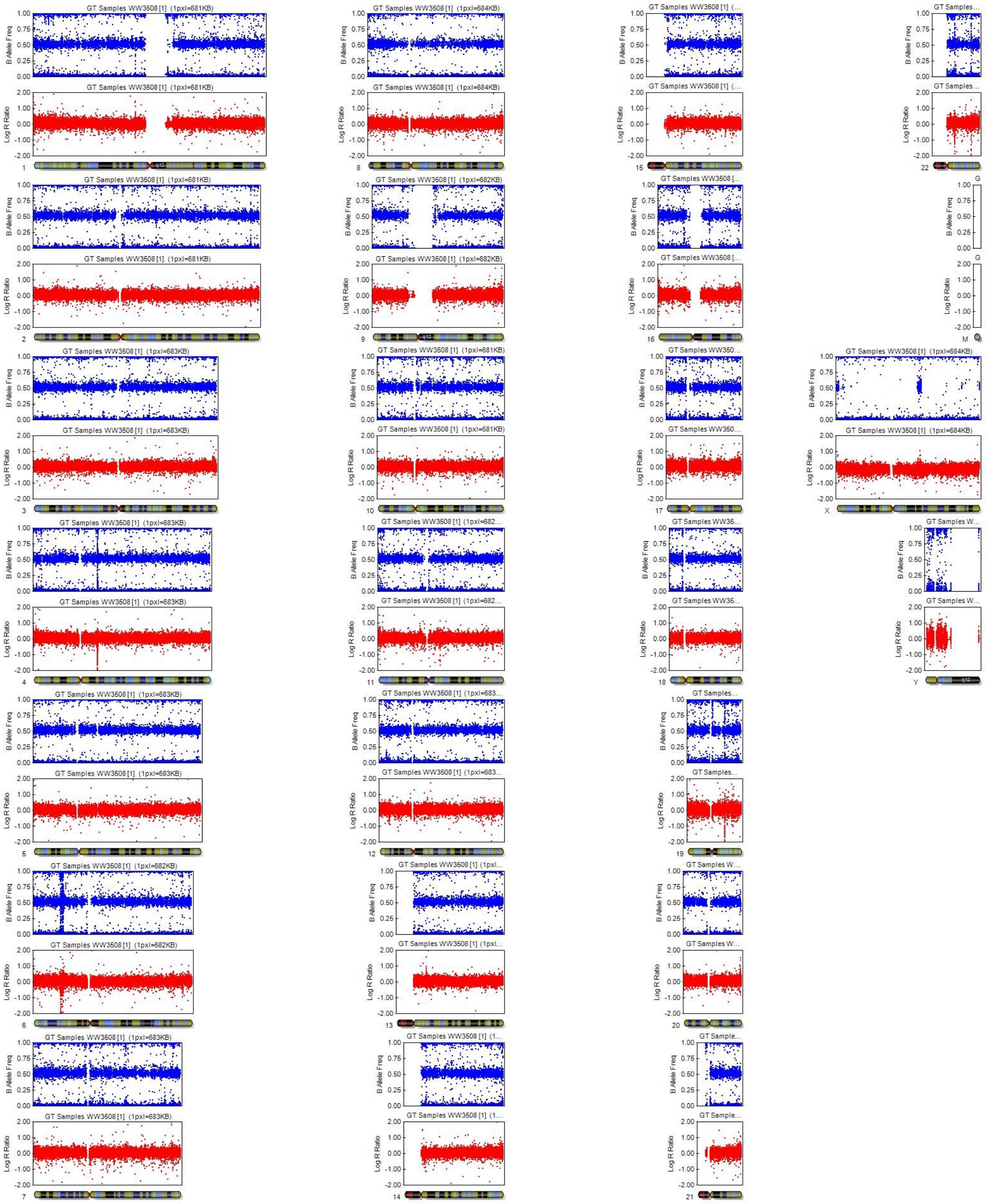

Supplementary Figure 5. Digital karyotyping by SNP array showing no chromosomal abnormalities in primary PBMCs.

5F iMSC digital karyotyping (*in vitro* culture for 1 week)

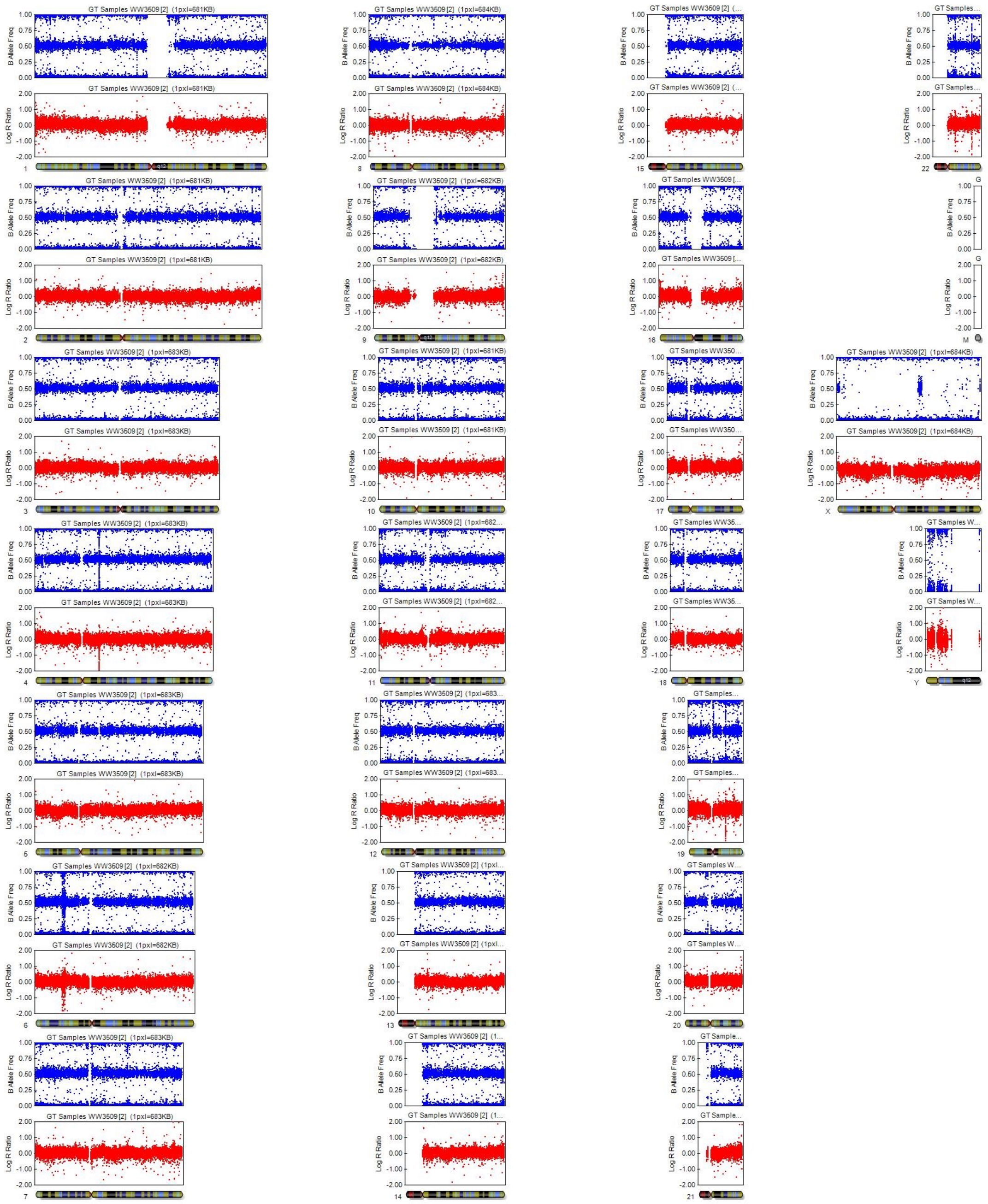

Supplementary Figure 6. Digital karyotyping by SNP array showing no chromosomal abnormalities in 5F iMSCs after one-week *in vitro* culture.

5F iMSC digital karyotyping (*in vitro* culture for 4 weeks)

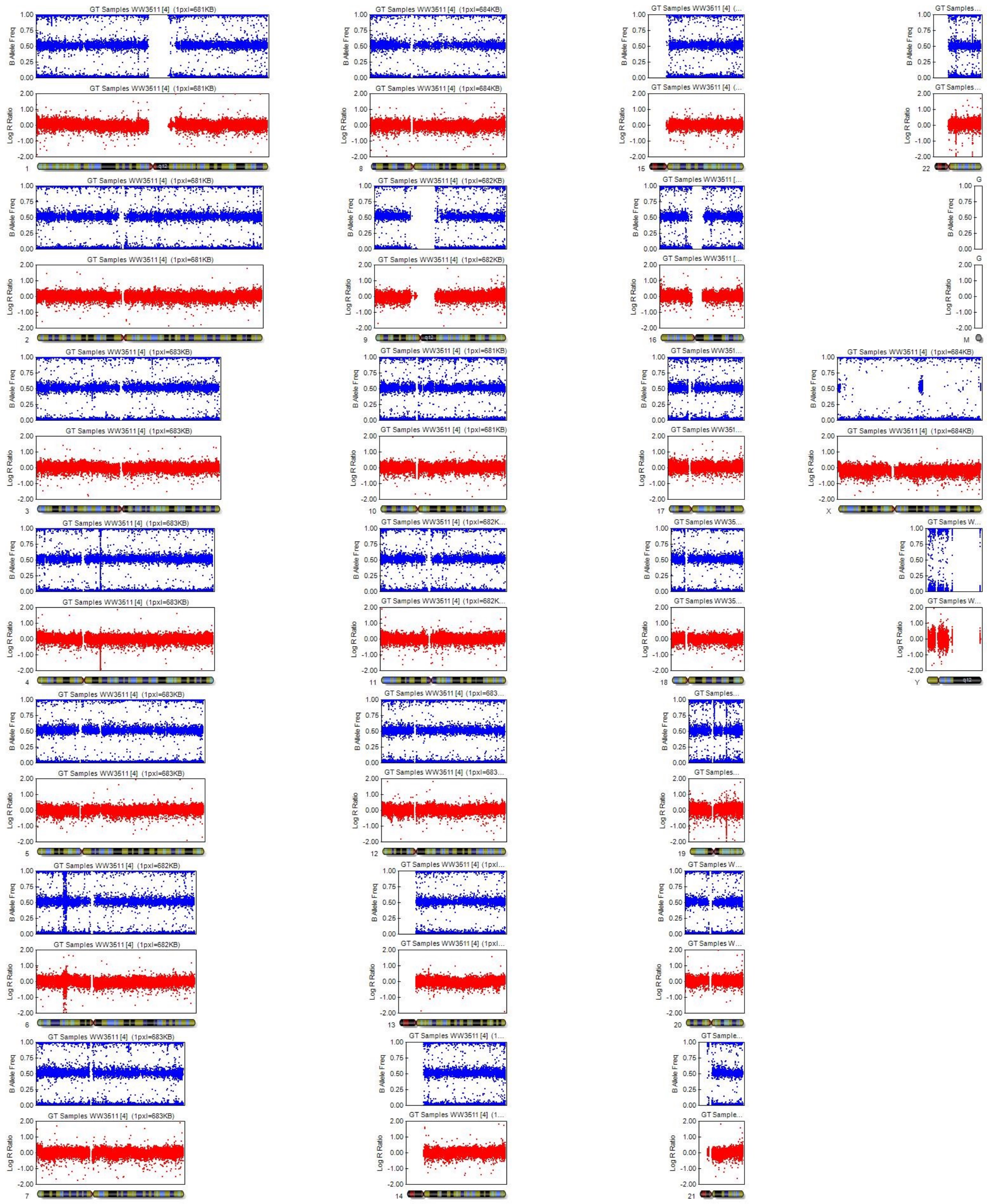

Supplementary Figure 7. Digital karyotyping by SNP array showing no chromosomal abnormalities in 5F iMSCs after four-week *in vitro* culture.

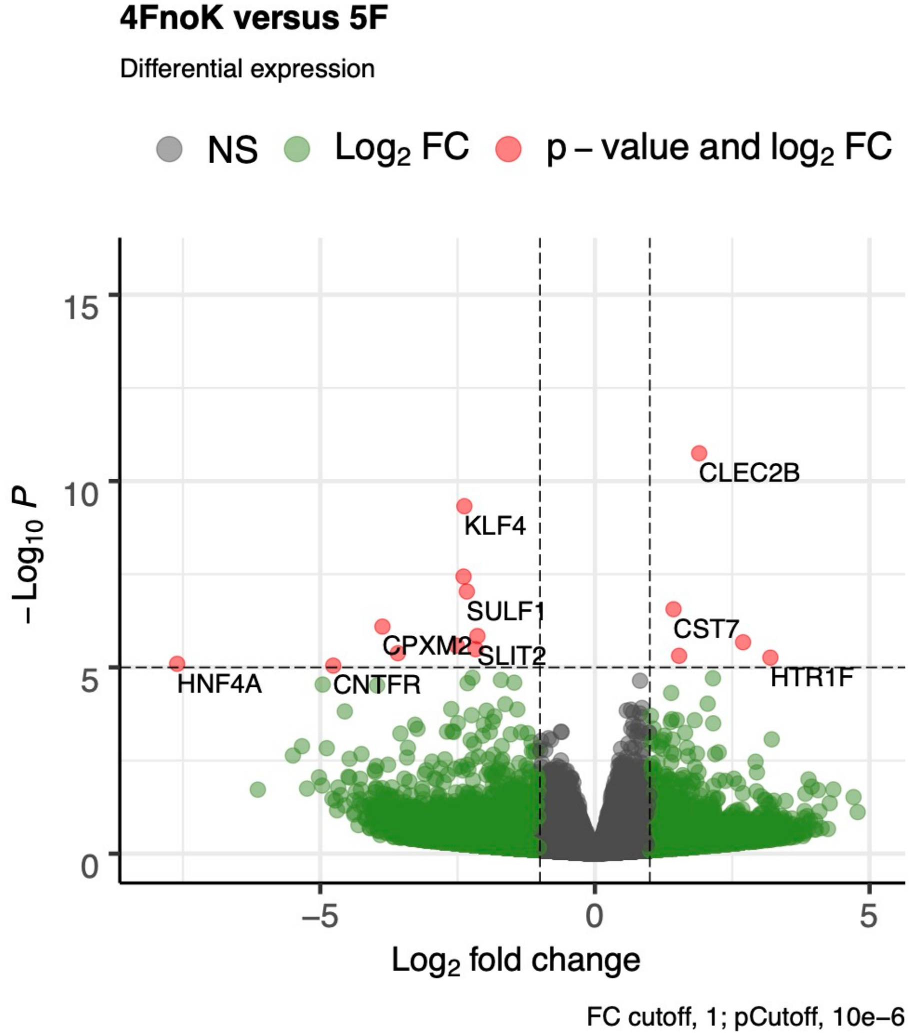

Supplementary Figure 8. Volcano plot showing the differential expressed genes between 5F iMSCs and 4FnoK iMSCs by RNA-seq (pCutoff = $10e^{-6}$, fold change > 2).

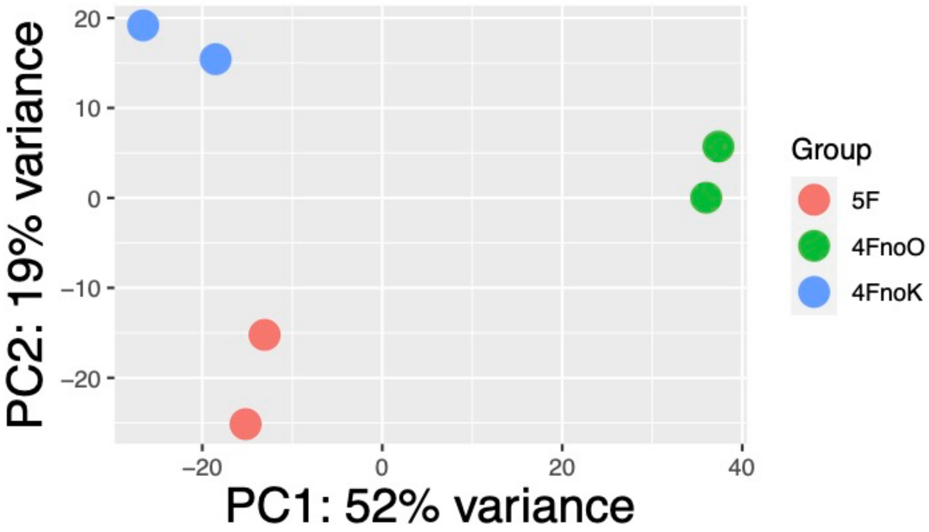

Supplementary Figure 9. Principal component analysis (PCA) using normalized ATAC-seq read counts from 5F, 4FnoO, and 4FnoK iMSCs. Each dot represents the chromatin accessibility profile of a biologically independent replicate.

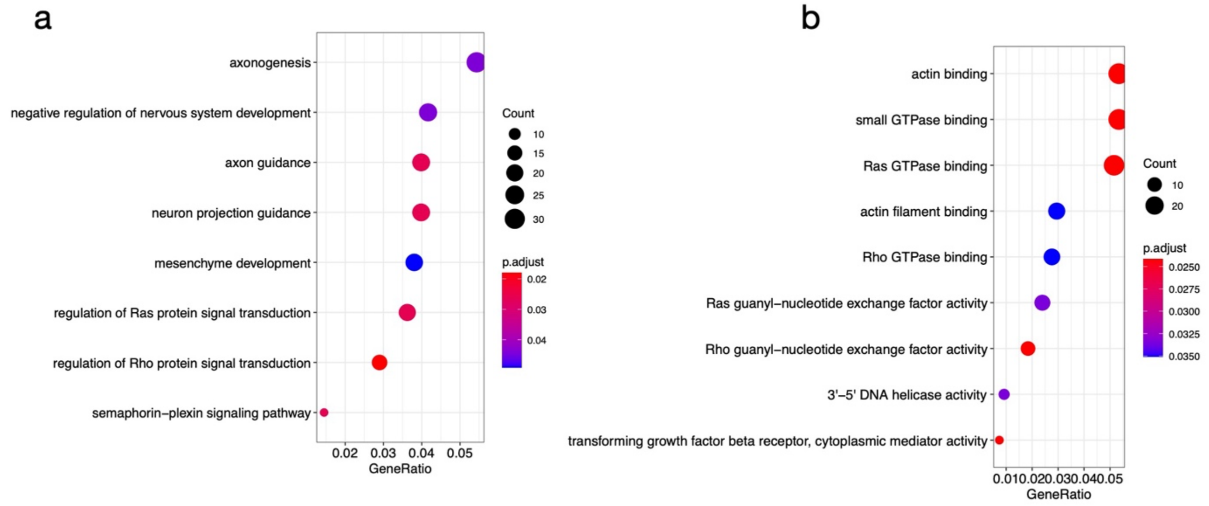

Supplementary Figure 10. Gene Ontology enrichment analysis of (a) biological processes and (b) cellular component for differential methylated genes between 5F iMSCs and 4FnoO iMSCs.

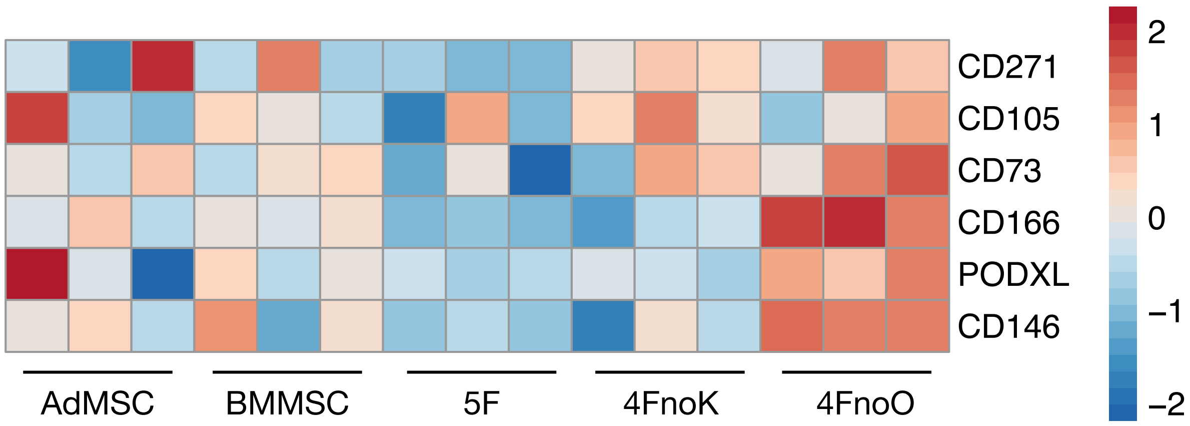

Supplementary Figure 11. The expression of typical MSC surface markers on primary MSCs and iMSCs. Heatmap showing the normalized gene count after $\log_2()$ transformation from RNA-seq data.

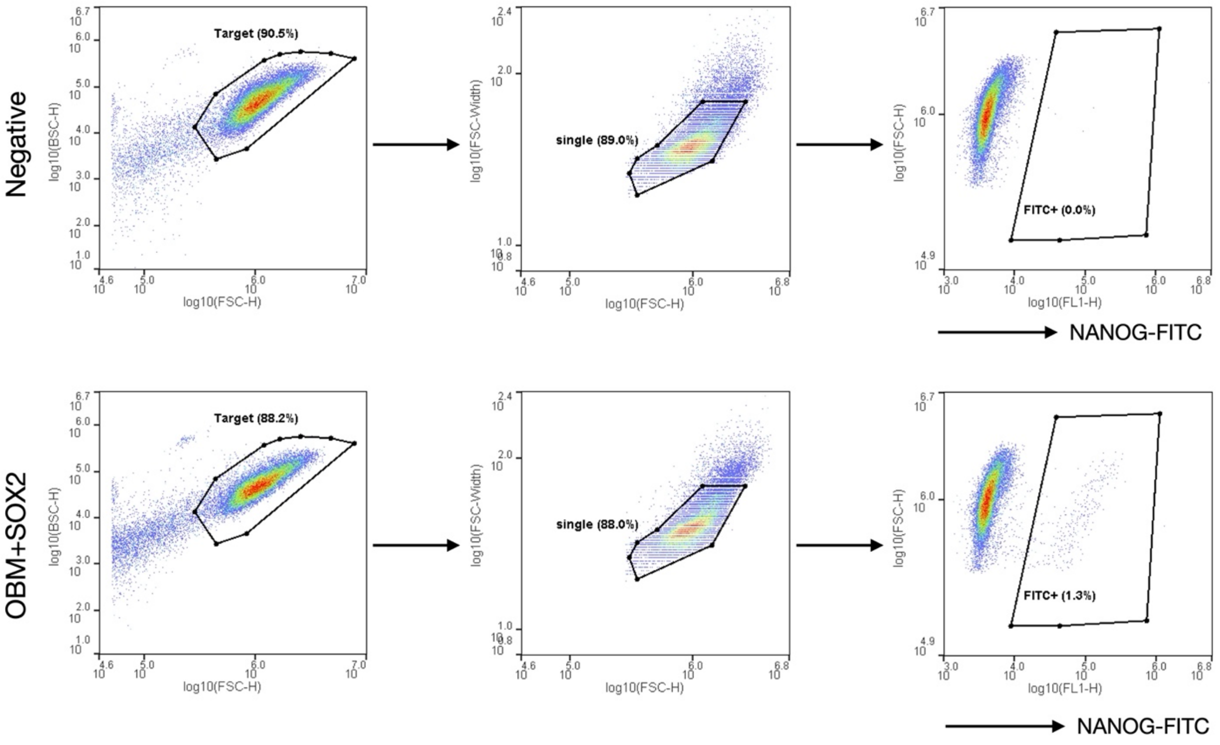

Supplementary Figure 12. Representative flow cytometry gating strategies. Scatterplots of forward scatter vs. side scatter showing live cell gating followed by single cell gating. Flow cytometric analysis of iMSCs showing that reprogramming in the presence of SOX2 resulted in 1-2% reprogrammed cells that were NANOG positive.

Supplementary Table 1. DEG overlapping DMG (4FnoO iMSCs vs. 5F iMSCs)

Symbol	Ensembl ID	BaseMean	log2Fold Change	lfcSE	pvalue	padj
	baseMean	log2Fold Change	lfcSE	stat	pvalue	padj
HPDL	138.376	-1.475	0.517	-2.850	0.004	0.050
COL24A1	276.043	-2.662	0.514	-5.176	0.000	0.000
F3	202.154	1.295	0.391	3.311	0.001	0.014
PALMD	228.902	-3.117	0.357	-8.727	0.000	0.000
IGSF3	245.693	-2.330	0.533	-4.371	0.000	0.000
ARHGAP30	486.515	1.701	0.304	5.589	0.000	0.000
LAMC2	78.083	-2.595	0.621	-4.179	0.000	0.001
PLXNA2	448.062	1.344	0.435	3.089	0.002	0.027
RNF144A-AS1	71.549	-1.541	0.427	-3.609	0.000	0.006
LINC01819	38.115	1.984	0.515	3.851	0.000	0.003
EPAS1	242.647	1.444	0.325	4.437	0.000	0.000
FHL2	921.563	-2.059	0.333	-6.181	0.000	0.000
SOWAHC	257.198	-2.658	0.503	-5.286	0.000	0.000
GALNT3	722.413	1.267	0.363	3.492	0.000	0.008
KCNE4	32.279	-2.559	0.755	-3.390	0.001	0.011
SEMA3B	295.663	1.109	0.315	3.523	0.000	0.007
NT5DC2	2021.290	-1.006	0.254	-3.963	0.000	0.002
WNT5A	843.097	-2.166	0.471	-4.598	0.000	0.000
EOGT	1021.022	-1.174	0.244	-4.814	0.000	0.000
FRMD4B	331.476	1.005	0.341	2.949	0.003	0.038
KLHL6	76.287	2.707	0.537	5.039	0.000	0.000
HAND2	64.516	-3.806	0.708	-5.374	0.000	0.000
PLEKHG4B	98.849	-3.373	0.448	-7.522	0.000	0.000
ENC1	1525.267	-1.603	0.393	-4.076	0.000	0.001
GFPT2	1023.661	-2.398	0.502	-4.778	0.000	0.000
SLC22A23	337.725	-2.005	0.425	-4.715	0.000	0.000
COL12A1	7985.834	1.534	0.227	6.769	0.000	0.000
GRIK2	241.869	2.738	0.414	6.618	0.000	0.000
TFPI2	1901.989	-5.173	1.038	-4.985	0.000	0.000
DUSP4	1406.671	-1.681	0.424	-3.967	0.000	0.002
ZFHX4	887.306	-4.892	0.437	-11.203	0.000	0.000
DENND3	195.507	1.619	0.315	5.145	0.000	0.000
ABCA1	624.513	-2.152	0.375	-5.743	0.000	0.000
OLFML2A	1693.597	2.396	0.310	7.724	0.000	0.000
PLPP7	45.761	-1.364	0.408	-3.345	0.001	0.013
COL5A1	15474.888	-1.520	0.444	-3.423	0.001	0.010
SLIT1	51.291	-3.700	0.784	-4.720	0.000	0.000
EPS8L2	425.508	1.340	0.256	5.224	0.000	0.000
NRXN2	20.903	-2.086	0.563	-3.702	0.000	0.004

ALDH3B1	372.252	1.394	0.347	4.016	0.000	0.001
NCAM1	488.208	-4.717	0.439	-10.733	0.000	0.000
PKNOX2	423.280	1.349	0.305	4.427	0.000	0.000
IRS2	680.866	-2.245	0.347	-6.471	0.000	0.000
GCH1	144.350	-1.405	0.358	-3.925	0.000	0.002
CCDC88C	298.078	1.424	0.333	4.277	0.000	0.001
CRIP2	522.616	1.329	0.275	4.834	0.000	0.000
THBS1	39420.693	2.289	0.347	6.587	0.000	0.000
C2CD4A	37.237	-4.439	1.010	-4.397	0.000	0.000
GPRC5B	185.104	-2.595	0.378	-6.870	0.000	0.000
NDRG4	139.239	-1.342	0.449	-2.990	0.003	0.035
ADORA2B	115.486	-1.081	0.356	-3.039	0.002	0.031
KRT16	16.151	-3.511	0.992	-3.541	0.000	0.007
ETV4	373.134	-2.161	0.362	-5.977	0.000	0.000
TANC2	1195.306	-1.103	0.200	-5.527	0.000	0.000
SOCS3	1034.345	-1.696	0.389	-4.363	0.000	0.000
TNFRSF11A	60.001	4.506	0.624	7.219	0.000	0.000
CTXN1	122.843	-1.640	0.369	-4.450	0.000	0.000
RDH8	8.616	-4.636	1.280	-3.623	0.000	0.005
DMKN	35.035	4.359	0.809	5.387	0.000	0.000
PPP1R13L	352.151	1.368	0.357	3.837	0.000	0.003
SLC8A2	589.649	-5.589	0.960	-5.823	0.000	0.000
PPFIA3	31.666	-1.663	0.495	-3.364	0.001	0.012
B4GALT5	1220.196	-1.037	0.186	-5.588	0.000	0.000
SALL4	201.538	-3.693	0.574	-6.429	0.000	0.000
LAMA5	1424.364	1.287	0.240	5.356	0.000	0.000
FAM227A	51.292	-1.093	0.363	-3.013	0.003	0.033
SLC9A7	1238.330	1.545	0.332	4.647	0.000	0.000

Supplementary Table 2. Primer sequences used for RT-qPCR analysis

Gene	Direction	Primer Sequence
ALP	Forward	5' -GAACAGGGCTTCTCAAGGTG -3'
	Reverse	5' -CAGTTCCGTCCCATTTCCTA -3'
SP7	Forward	5' -GGCACAAAGAAGCCGTACTC -3'
	Reverse	5' -CACTGGGCAGACAGTCAGAA -3'
RUNX2	Forward	5' -TTTGCACTGGGTCATGTGTT -3'
	Reverse	5' -TGGCTGCATTGAAAAGACTG -3'
LPL	Forward	5' -ATTTGCCCTAAGGACCCC -3'
	Reverse	5' -ATGACAGGTAGCCACGGAC -3'
FABP4	Forward	5' -TACTGGGCCAGGAATTTGAC -3'
	Reverse	5' -GTGGAAGTGACGCCTTTCAT -3'
SOX9	Forward	5' -GACTTCCGCGACGTGGAC -3'
	Reverse	5' -GTTGGGCGGCAGGTACTG -3'
ACAN	Forward	5' -CGCTACTCGCTGACCTTT -3'
	Reverse	5' -GCTCATAGCCTGCTTCGT -3'
COL10A1	Forward	5' -CAGGCATAAAAGGCCCACTA -3'
	Reverse	5' -AGGACTTCCGTAGCCTGGTT -3'
COL1A1	Forward	5' -GCCATCAAAGTCTTCTGC -3'
	Reverse	5' -ATCCATCGGTCATGCTCT -3'
ACTB	Forward	5' -TCGTGCGTGACATTAAGGAG -3'
	Reverse	5' -GGCAGCTCGTAGCTCTTCTC -3'

Supplementary Table 3. RNA-seq, ATAC-seq, and RRBS libraries sequencing summary

Library_ID	Group	Sequence length	Reads (M)	Application	PBMC Donor	Platform
CFG2295	5F	100x1 bp	24.8	RNA-seq	PM18	HiSeq4000
CFG2296	4FnoO	100x1 bp	31.6		PM18	HiSeq4000
CFG2334	4FnoK	100x1 bp	24.7		PM18	HiSeq4000
CFG2336	5F	100x1 bp	26.2		PM10	HiSeq4000
CFG2337	4FnoK	100x1 bp	22.6		PM10	HiSeq4000
CFG2338	4FnoO	100x1 bp	27.3		PM10	HiSeq4000
RS193	5F	150x1 bp	24.1		PM9	HiSeq4000
RS194	4FnoO	150x1 bp	30.5		PM9	HiSeq4000
RS195	4FnoK	150x1 bp	27.5		PM9	HiSeq4000
CFG2357	5F	75x2 bp	60.6	ATAC-seq	PM10	NextSeq550
CFG2358	4FnoO	75x2 bp	64.6		PM10	NextSeq550
CFG2359	4FnoK	75x2 bp	71		PM10	NextSeq550
CFG2360	5F	75x2 bp	69		PM9	NextSeq550
CFG2361	4FnoO	75x2 bp	76.4		PM9	NextSeq550
CFG2363	4FnoK	75x2 bp	76.4		PM9	NextSeq550
CFG2351	5F	100x1 bp and 75x1 bp	29.5	RRBS	PM18	HiSeq4000
CFG2352	4FnoO	100x1 bp and 75x1 bp	32.6		PM18	HiSeq4000
CFG2353	4FnoK	100x1 bp and 75x1 bp	29.1		PM18	HiSeq4000
CFG2354	5F	100x1 bp and 75x1 bp	30.2		PM10	HiSeq4000
CFG2355	4FnoO	100x1 bp and 75x1 bp	30.5		PM10	HiSeq4000
CFG2356	4FnoK	100x1 bp and 75x1 bp	30.1		PM10	HiSeq4000
