# Supplementary material for: Reprogramming of human peripheral blood mononuclear cells into induced mesenchymal stromal cells using non-integrating vectors
Source: Commun Biol. 2023 Apr 11;6:393. doi: 10.1038/s42003-023-04737-x (PMC10090171; doi:10.1038/s42003-023-04737-x)
Supplement: Supplementary file 3 — Description of Additional Supplementary Files [file 42003_2023_4737_MOESM3_ESM.pdf]

Description of Additional Supplementary Files

File name: Supplementary Data 1

Description: The source data for figure 1c

File name: Supplementary Data 2

Description: The source data for figure 2b

File name: Supplementary Data 3

Description: The source data for figure 2f and supplementary figure 4a

File name: Supplementary Data 4

Description: The source data for figure 3a

File name: Supplementary Data 5

Description: The source data for figure 3d-3f

File name: Supplementary Data 6

Description: The source data for Supplementary figure 4b

File name: Supplementary Data 7

Description: DEGs of 4FnoO iMSCs vs. 5F iMSCs.

File name: Supplementary Data 8

Description: The source data for figure 5e, 6f, and 7d

File name: Supplementary Data 9

Description: The source data for figure 6a and 6b

File name: Supplementary Data 10

Description: DMCs of 4FnoO iMSCs vs. 5F iMSCs
