# Supplementary material for: Reprogramming of human peripheral blood mononuclear cells into induced mesenchymal stromal cells using non-integrating vectors
Source: Commun Biol. 2023 Apr 11;6:393. doi: 10.1038/s42003-023-04737-x (PMC10090171; doi:10.1038/s42003-023-04737-x)
Supplement: Supplementary file 14 — Reporting Summary [file 42003_2023_4737_MOESM14_ESM.pdf]

nature portfolio

Corresponding author(s): Charles Wang

Last updated by author(s): 1/31/2023

Reporting Summary

Nature Portfolio wishes to improve the reproducibility of the work that we publish. This form provides structure for consistency and transparency in reporting. For further information on Nature Portfolio policies, see our Editorial Policies and the Editorial Policy Checklist.

Statistics

For all statistical analyses, confirm that the following items are present in the figure legend, table legend, main text, or Methods section.

n/a	Confirmed	
☐	☒	The exact sample size (*n*) for each experimental group/condition, given as a discrete number and unit of measurement
☐	☒	A statement on whether measurements were taken from distinct samples or whether the same sample was measured repeatedly
☐	☒	The statistical test(s) used AND whether they are one- or two-sided<br>*Only common tests should be described solely by name; describe more complex techniques in the Methods section.*
☐	☒	A description of all covariates tested
☒	☐	A description of any assumptions or corrections, such as tests of normality and adjustment for multiple comparisons
☐	☒	A full description of the statistical parameters including central tendency (e.g. means) or other basic estimates (e.g. regression coefficient) AND variation (e.g. standard deviation) or associated estimates of uncertainty (e.g. confidence intervals)
☐	☒	For null hypothesis testing, the test statistic (e.g. *F*, *t*, *r*) with confidence intervals, effect sizes, degrees of freedom and *P* value noted<br>*Give P values as exact values whenever suitable.*
☒	☐	For Bayesian analysis, information on the choice of priors and Markov chain Monte Carlo settings
☒	☐	For hierarchical and complex designs, identification of the appropriate level for tests and full reporting of outcomes
☒	☐	Estimates of effect sizes (e.g. Cohen's *d*, Pearson's *r*), indicating how they were calculated

Our web collection on statistics for biologists contains articles on many of the points above.

Software and code

Policy information about availability of computer code

Data collection	We used many publicly available algorithms and packages for the RNA-seq, ATAC-seq and RRBS mapping, differential analysis, genome annotation,etc., which were cited properly in the manuscript. This paper did not produce original code.
Data analysis	The details of methods used were described in the Methods section.

For manuscripts utilizing custom algorithms or software that are central to the research but not yet described in published literature, software must be made available to editors and reviewers. We strongly encourage code deposition in a community repository (e.g. GitHub). See the Nature Portfolio guidelines for submitting code & software for further information.

Data

Policy information about availability of data

All manuscripts must include a data availability statement. This statement should provide the following information, where applicable:

- Accession codes, unique identifiers, or web links for publicly available datasets
- A description of any restrictions on data availability
- For clinical datasets or third party data, please ensure that the statement adheres to our policy

All the sequencing data have been uploaded to the NCBI GEO (Gene Expression Omnibus) under accession number GSE193201.

Human research participants

Policy information about studies involving human research participants and Sex and Gender in Research.

Reporting on sex and gender	N/A
Population characteristics	N/A
Recruitment	N/A
Ethics oversight	N/A

Note that full information on the approval of the study protocol must also be provided in the manuscript.

Field-specific reporting

Please select the one below that is the best fit for your research. If you are not sure, read the appropriate sections before making your selection.

☒ Life sciences ☐ Behavioural & social sciences ☐ Ecological, evolutionary & environmental sciences

For a reference copy of the document with all sections, see nature.com/documents/nr-reporting-summary-flat.pdf

Life sciences study design

All studies must disclose on these points even when the disclosure is negative.

Sample size	Sample size was reported in the manuscript.
Data exclusions	No data were excluded from the analysis.
Replication	At least three biologically independent experiments for cell culture and flow cytometry experiments were performed. For sequencing data, PBMCs from two to three individual subjects were used as biological replicates.
Randomization	n/a
Blinding	n/a

Reporting for specific materials, systems and methods

We require information from authors about some types of materials, experimental systems and methods used in many studies. Here, indicate whether each material, system or method listed is relevant to your study. If you are not sure if a list item applies to your research, read the appropriate section before selecting a response.

Materials & experimental systems

n/a	Involved in the study	
☐	☒	Antibodies
☒	☐	Eukaryotic cell lines
☒	☐	Palaeontology and archaeology
☒	☐	Animals and other organisms
☒	☐	Clinical data
☒	☐	Dual use research of concern

Methods

n/a	Involved in the study	
☒	☐	ChIP-seq
☐	☒	Flow cytometry
☒	☐	MRI-based neuroimaging

Antibodies

Antibodies used	CD34(ThermoFisher/eBioscience, 17-0349-42, clone 4H11)<br>CD45(ThermoFisher/eBioscience, 11-0459-42, clone HI30)<br>CD29(ThermoFisher/eBioscience, 17-0299-42, clone TS2/16)<br>CD73(ThermoFisher/eBioscience, 17-0739-42, clone AD2)<br>CD90(ThermoFisher/eBioscience, 12-0909-42, clone 5E10)<br>CD166(ThermoFisher/eBioscience, 12-1668-42, clone 3A6)<br>TRA-1-60(ThermoFisher/eBioscience, 12-8863-82, clone TRA-1-60)

NANOG(ThermoFisher, MA1-017-D488, clone 23D2-3C6)
OCT3/4(ThermoFisher, 53-5841-82, clone EM92)

Validation: www.thermofisher.com

Flow Cytometry

Plots

Confirm that:

☒ The axis labels state the marker and fluorochrome used (e.g. CD4-FITC).

☒ The axis scales are clearly visible. Include numbers along axes only for bottom left plot of group (a 'group' is an analysis of identical markers).

☒ All plots are contour plots with outliers or pseudocolor plots.

☒ A numerical value for number of cells or percentage (with statistics) is provided.

Methodology

Sample preparation	Cells were dissociated with Accutase (Innovative Cell Technologies, Inc., CA) and stained with specific antibodies.
Instrument	BD FACSAria II flow cytometer and Nanocellect WOLF cell sorter
Software	BD FACSDiva Software and WOLF 2.2.200
Cell population abundance	The purity of gated populations was generally in the range of 1-99%
Gating strategy	Gate on fsc vs. ssc was set to include all cell populations, but excluding debris and dead cells as shown in Supplementary Fig.12.

☒ Tick this box to confirm that a figure exemplifying the gating strategy is provided in the Supplementary Information.
